# Short-Term Entropy of Signal Energy Used for Effective Detecting of Weak Gunshots in Noisy Environments

**DOI:** 10.3390/s24154933

**Published:** 2024-07-30

**Authors:** Milan Sigmund

**Affiliations:** Department of Radio Electronics, Faculty of Electrical Engineering and Communication, Brno University of Technology, Technicka 3082/12, 61600 Brno, Czech Republic; sigmund@vut.cz

**Keywords:** gunshot detection, weak gunshots, short-term energy entropy, noisy environment, security and surveillance

## Abstract

Conventional gunshot detection systems can quickly and reliably detect gunshots in the area where the acoustic sensors are placed. This paper presents the detection of weak hunting gunshots using the short-term entropy of signal energy computed from acoustic signals in an open natural environment. Our research in this field was primarily aimed at detecting gunshots fired at close range with the usual acoustic intensity to protect wild elephants from poachers. The detection of weak gunshots can extend existing detection systems to detect more distant gunshots. The developed algorithm was optimized for the detection of gunshots in two categories of the surrounding sounds, short impulsive events and continuous noise, and tested in acoustic scenes where the power ratios between the weak gunshots and louder surroundings range from 0 dB to −14 dB. The overall accuracy was evaluated in terms of recall and precision. Depending on impulsive or noise sounds, binary detection was successful down to −8 dB or −6 dB; then, the efficiency decreases, but some very weak gunshots can still be detected at −13 dB. Experiments show that the proposed method has the potential to improve the efficiency and reliability of gunshot detection systems.

## 1. Introduction

The sound produced when fired is determined by the mechanical parameters of the weapon, such as the bullet caliber and barrel length, as well as the characteristics of the ammunition used. However, the measured sound may be influenced more or less by the acoustics at the place where the gunshot has been taken, depending on the shape of the natural relief, density of the surrounding vegetation, size and material of the surrounding buildings, and weather. More details about the effects of atmospheric factors (temperature, relative humidity, wind, and ground surface) to spread sound from gunshots can be found in [1]. For example, the propagation of the acoustic wave at night is slightly different due to the different temperature. The process of sound absorption in the air related to temperature and humidity is explained in [2].

Gunshots are very short, high-intensive impulse sounds. In real-world recordings, a muzzle blast typically lasts for 3 to 5 ms depending on the physical environment [3]. In an anechoic environment, the muzzle blast duration for common firearm types is less than 2 ms [4]. Details for determining the muzzle blast duration can be found in [4]. The sound pressure level (SPL) generated by small-caliber rifles, shotguns, and large-caliber handguns range from a 132 dB up to 172 dB peak SPL for high-powered firearms [5]. For example, the unweighted peak SPL produced by AK-15 rifle shots was measured to be 168 dB at a distance of 1.8 m and 150 dB at 4.3 m [5]. In fact, the SPL varies with the distance from the gunshot decreasing by approx. 6 dB for every doubling of the distance [3], and depends upon the direction relative to the barrel axis [6]. Due to the high SPL, some gunshots can be heard under normal atmospheric conditions at a distance of 2–3 km.

Automatic gunshot detection systems are developed to continuously monitor locations where a gunshot could potentially be heard, even when no gunshot should occur there. Depending on the monitored environment, gunshot detections fall into one of three categories, namely:

- detection in an open nature environment [7,8];
- detection in an urban environment [9,10,11];
- detection in an indoor environment [12,13].

The type of firearms used usually varies depending on the purpose and, therefore, the place of use. While short, small-caliber weapons (pistols) are most often used indoors, in the open space, longer, larger-caliber weapons (rifles) or automatic weapons are used. An important aspect when developing a system for indoor, urban, or open natural spaces is the possible acoustic scene around the microphones. Any sounds similar to gunshots, in particular, play a significant role. The goal of gunshot detection is not only to correctly detect every gunshot, but also to ignore sounds that are not gunshots. In each type of environment, there are some common sounds that may resemble a gunshot, e.g., a door slamming indoors in a sudden draft, a car door slamming by a person in the street, wood chopping in the countryside, church bells ringing, etc. The main purpose of automatic gunshot detection is, on the one hand, to improve the safety of people (the prevention of violent crime and the investigation of crime scenes), and, on the other hand, to help reveal illegal poaching in wildlife areas (catching poachers).

Of course, many gunshot detection techniques are required in military applications. Special systems for the detection and localization of gunshots are used in war operations to neutralize snipers. Such systems must operate in real time or near real time. A mobile shooter detection system suitable for installation on military patrol vehicles is presented in [14]. Some information on the detection of shots from a diverse range of military weapons can be found, for example, in [15]. Presentations of military systems are usually limited to an overall description of the entire system, focusing on the main advantages from a military perspective. In this article, gunshots in military scenarios are not taken into account.

Our research into gunshot detection is focused on acoustic observation in open nature. A special gunshot detection application has been initiated by the Save Elephants society to protect elephants against poachers in Central Africa. Some wild elephants today wear collars that are equipped with a GPS module to track the movements of elephant herds in the wild. The collars can be additionally equipped with an autonomous gunshot detection module that sends a wireless alarm signal along with geographic co-ordinates of the gunshot location. In this case, real-time information about poaching events will be sent to the control center and alert anti-poaching teams. The guard team closest to the incident can then intervene immediately. An overview of the current monitoring of elephants in the wild can be found, for example, in [16].

In our current work, we focus on gunshot sounds that have a low signal energy. The reason for the low energy is usually the large distance between the acoustic source and the sensor. Since low-energy sounds are perceived as quiet or barely audible, such gunshots will be termed as “weak gunshots” hereafter. The detection of very weak gunshots in various noisy acoustic environments is not routinely performed in existing systems and can, therefore, be considered as an added value extending the applicability of standard acoustic monitoring systems.

The rest of the article is organized as follows: In the next section, a brief overview of the current state of the art is given along with important related works. Section 3 introduces the gunshot sound signal, including its propagation, describes the sound data used in the experiments, and defines the short-term entropy of the signal energy as an effective feature for weak gunshot detection. Experimental results achieved in tests with both short impulsive acoustic events and continuous noise are summarized in Section 4. Finally, Section 5 concludes this paper.

## 2. Related Works

In general, weak signals cover various specific waveforms that need to be dealt with in vast areas of signal processing such as wireless communication, biomedical sensors, radar, sonar, etc. There is no uniform framework for exploiting weak signals. The methods used differ depending on the target application. In [17], a method is proposed for the detection of underwater weak signals in the complex sea background and the extraction of their frequencies. The detection of weak acoustic signals using stochastic resonance is described in [18]. Study [19] presents an analysis for the accurate parameter estimation of weak radiofrequency signals in a process with multiple simultaneous communication signals. Many recommendations for literature on weak signals in space exploration can be found in [20]. In an industrial application, weak signal recognition in the drilling process is solved in [21]. An interdisciplinary review on weak signal detection is given, for example, in [22]. In practical research, the accuracy of measuring and recording weak signals plays a key role. Paper [23] offers a review of precision lock-in amplifiers for applications in various weak signal areas.

In the last two decades, various computer algorithms have been developed to detect dangerous sounds including gunshots, but high-performance algorithms are usually limited for use under specific conditions. Acoustic systems for the protection of tropical forests against potential destroying activities usually combine the detection of gunshots and chainsaws [24]. For security monitoring in urban areas, the detection of gunshots is often combined with the detection of glass breaking; see, for example, [25]. A generic emergency detection system based on sounds produced in an environment, where the gunshots are one of the observed emergency classes, is proposed in [26]. In general, gunshot detection algorithms use both specially developed features for gunshots, as presented, for example, in paper [27], and proven universal features for classifying many types of signals, such as spectrograms created using the Fourier transform [28] or wavelet transform [29]. The most common features used in acoustic signal processing are mel-frequency cepstral coefficients (MFCCs). These coefficients are based on the non-linear and masking psychoacoustic characteristics of human hearing. They are generally effective for recognizing sounds that can be distinguished by hearing, which also applies to distinctive gunshots. A detailed analysis of the first 40 MFCCs, as well as their differentiation Δ and double differentiation ΔΔ extracted from gunshots, is given in [30]. An efficient modification of MFCCs for gunshot detection can be found in [31].

Most research publications present results obtained using user-friendly programming environments such as MATLAB or Python. Only few studies provide experience gained in hardware design and implementation. An attempt to implement and test gunshot detection on a field-programmable gate array (FPGA) is introduced in [32]. Paper [33] deals with some aspects of practical implementation in real time on a signal processor. A fully functional gunshot detection and localization system implemented as a prototype based on a micro-controller from the family SMT32 is presented in [34].

Almost all the approaches developed are for loud gunshots, sometimes with added low ambient noise. Research on the detection of weak gunshots is still not sufficiently addressed. To our knowledge, no previous publication has specifically focused on such a topic. Only one study also includes gunshots that have an energy lower than the energy of the background sounds [35]. Our results are compared with this study in Section 4.

## 3. Materials and Methods

### 3.1. Sound Signal of Gunshots

Gunshots can be considered as impulses spreading into the surroundings in the atmosphere. Important parameters characterizing the propagation of gunshots through the air [36] are Mach number

*M* = *v*/*c* (1)

and Mach angle

**Figure 1:**
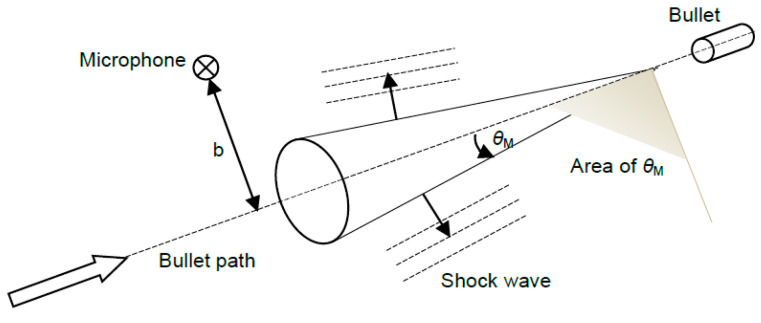
Bullet shock wave propagation.

*θ*_M_ = arcsin(1/*M*), (2)

where *v* is the bullet’s speed and *c* stands for the local speed of sound (typically *c* = 343 m/s at 20 °C). As can be seen, the speed of the bullet *v* determines the direction of the shock wave propagation (assuming that *c* is constant in time and space of the gunshot). In case of a very fast bullet, *M* is large, *θ*_M_ becomes small, and the shock wave propagates nearly perpendicularly to the path of the bullet. On the contrary, if the bullet’s speed is only slightly higher than the speed of the sound, *M* is approximately equal to one, *θ*_M_ is almost 90°, and the shock wave propagates nearly parallel to the path of the bullet. The geometry of the supersonic shock wave propagation is depicted in Figure 1. The shaded area indicates possible range of the Mach angle.

**Figure 2:**
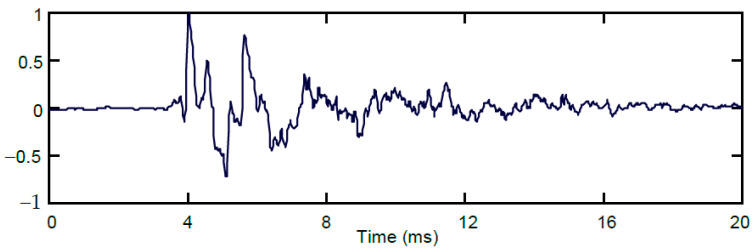
Waveform of a single gunshot.

Mach number also partially determines the shape of the N-wave, the typical initial part of gunshot signal. The peak pressure maximum *p*_max_ and the time interval *T* between the positive and negative peaks in the N-wave are given by equations

(3) $$p_{max}=\frac{0.53P_{0}\;{\left(M^{2}-1\right)}^{1/8}}{b^{3/4}}\;\;\frac{d}{l^{1/4}}$$

(4) $$T\approx 1.82\;\left(\frac{d}{c}\right)\;{\left(\frac{Mb}{l}\right)}^{1/4},$$

where *P*_0_ is ambient pressure, *d* is the bullet diameter, *l* is the bullet length, and *b* is the distance between the bullet’s path and the microphone (measured as perpendicular distance). In free space, the sound energy of the gunshots does not spread uniformly in all directions, but most acoustic energy is concentrated near the barrel axis. Many details on the waveform shape and its distortion during shock propagation can be found in [37]. A typical gunshot sound waveform is shown in detail in Figure 2.

**Figure 3:**
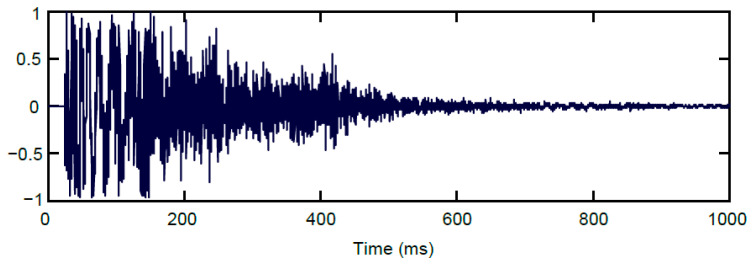
Example of a synthetic gunshot intended for adventure movies.

In addition to real gunshots, we have also investigated synthetic gunshots intended for entertainment productions such as movies, computer games, etc. Such sounds are perceived by lay listeners as gunshots, but, in contrast to real gunshots, they usually have a different waveform. Especially in Western adventure movies (cowgirls), used synthetic gunshots have a longer duration and sound more pleasant than real gunshots. An example of such a gunshot is illustrated in Figure 3.

### 3.2. Sound Data Used

For effective training and testing, it is important to have a wide range of audio data representing not only the gunshots, but also ambient sounds typically found in the monitored soundscape. Useful information on various audio databases focusing on many audio events, annotation tools, and audio management tools can be found in [38].

We used recordings from our database named Gunshot Detection in Open Nature (GUDEON), which was recently specially created for experimental research on poacher shooting detection. The database contains selected sounds of interest collected from several available sound sources, as well as our own recordings. The gunshot category includes 1304 sounds of hunting weapons and military assault rifles used by poachers for hunting. Since the gunshots were recorded while firing outdoors in an open area, the audio signals do not contain any echo. The non-gunshot category covers wildlife sounds (dogs barking, elephants snorting, and trumpet calls), vehicle sounds (off-road cars, car horns, and low-flying helicopters), human screams, etc.—a total of 983 sounds. All audio signals from GUDEON used in the following tests were in WAV audio format, monophonic, 16-bit, and with a 44.1 kHz sampling rate. The entire database is introduced in [39].

**Figure 4:**
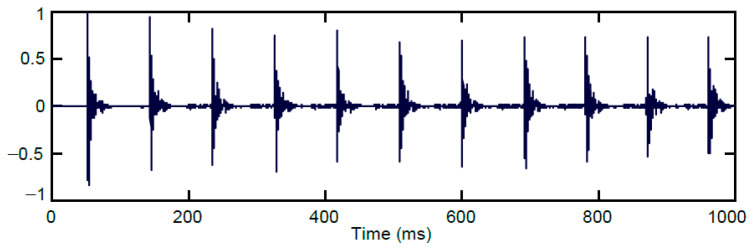
Example of short burst from an AK-47.

The largest group of sounds of the same type is represented by gunshots from the AK-47 assault rifle. This is not typically a hunting weapon, but most frequently used by poachers in Central Africa. It can produce two kinds of gunshots by either firing in single shot mode or rapid burst mode. Figure 4 shows a sequence of gunshots fired automatically in a burst. The distance between individual gunshots in one burst is approx. 90 ms. The initial gunshot usually has the maximum amplitude in the burst.

### 3.3. Method Based on Entropy of Energy

The standard gunshot processing techniques begin by segmenting the audio signal into short segments. Then, in each segment, the relevant signal characters are extracted and collected in a feature vector. This principle is taken from the analysis of speech and music signals, where it has proven very effective. The algorithms used to obtain the feature vectors can be applied in both the time and frequency domain.

In our research, we searched for efficient features to detect weak gunshots in a mixture of audio events. Practical experiments show very promising short-term entropy of signal energy. It can be interpreted as a feature expressing unexpected sudden changes in the energy level of the observed audio signal. First, the short-term energy of the *j*-th segment of the discrete-time sound signal *s*(*n*) containing *n* = 1,…, *N* samples is computed

(5) $$E(j)=\sum_{n=1}^{N}{\left|s(n)\right|}^{2}.$$

In the next step, each segment is divided into *K* sub-segments of uniform length equal to *Nsub* = *N*/*K* points, and, for each sub-segment *k*, the sub-segment energy is computed

(6) $$Esub_{k}(j)=\sum_{n=1}^{Nsub}{\left|s(n)\right|}^{2},$$

where *n* = 1 is the beginning of the *k*-th sub-segment. Then, the energies of all sub-segments are normalized by the energy of the entire segment

(7) $$e_{k}(j)=\frac{Esub_{k}(j)}{E(j)}.$$

Finally, the entropy of energy in the *j*-th segment is computed from the sequence $e_{k}(j)$

(8) $$H(j)=-\sum_{k=1}^{K}e_{k}(j)\ln\left[e_{k}(j)\right].$$

Experiments also show that the performance of the entropy *H*(*j*) can be further improved by appropriately weighting the audio signal using a short-term window. In order to estimate the effect of signal windowing on the entropy, the signal in each segment as well as in each sub-segment was windowed with two types of windows, namely, rectangular window

(9) r(n)=1,

and Hamming window

(10) h(n)=0.54−0.46cos(2πn/N).

**Figure 5:**
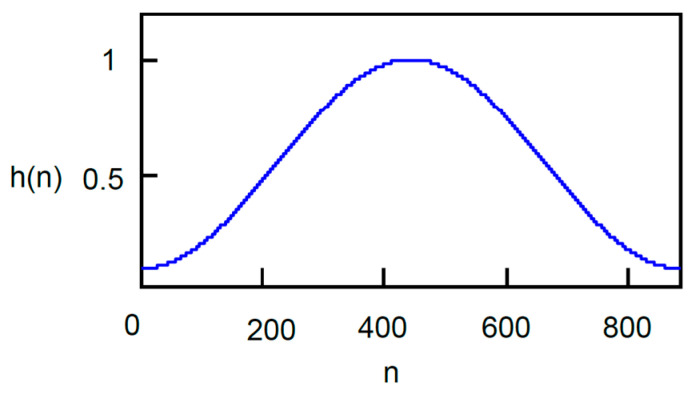
Hamming window in time domain for window length *N* = 880.

In the window Functions (9) and (10), the variable *n* ranges according to the segmentation from 1 to *N* or from 1 to *Nsub*. Outside the window range, i.e., n∉〈1,N〉 or n∉〈1,Nsub〉, the window values are zeros. The window function *h*(*n*), which tapers the signal amplitude toward both edges of the segment, is symmetric around the window center *N*/2, as can be seen in Figure 5. The rectangular window is the simplest window that requires minimal computation. The Hamming window is widely used window type in audio signal processing. This window highlights spectral lines, but reduces the signal energy at the edges of the window. Weak gunshots have low energy and this is further reduced by the Hamming window. It is useful, for example, in the processing of speech signals or music signals. The Hamming window was also used in practical testing of the proposed approach as a representative of tapered windows to show that, in this case, a rectangular window should be preferred not for its simplicity but for effectivity. As can be seen later in Section 4.2, the theoretical assumption was borne out experimentally.

**Figure 6:**
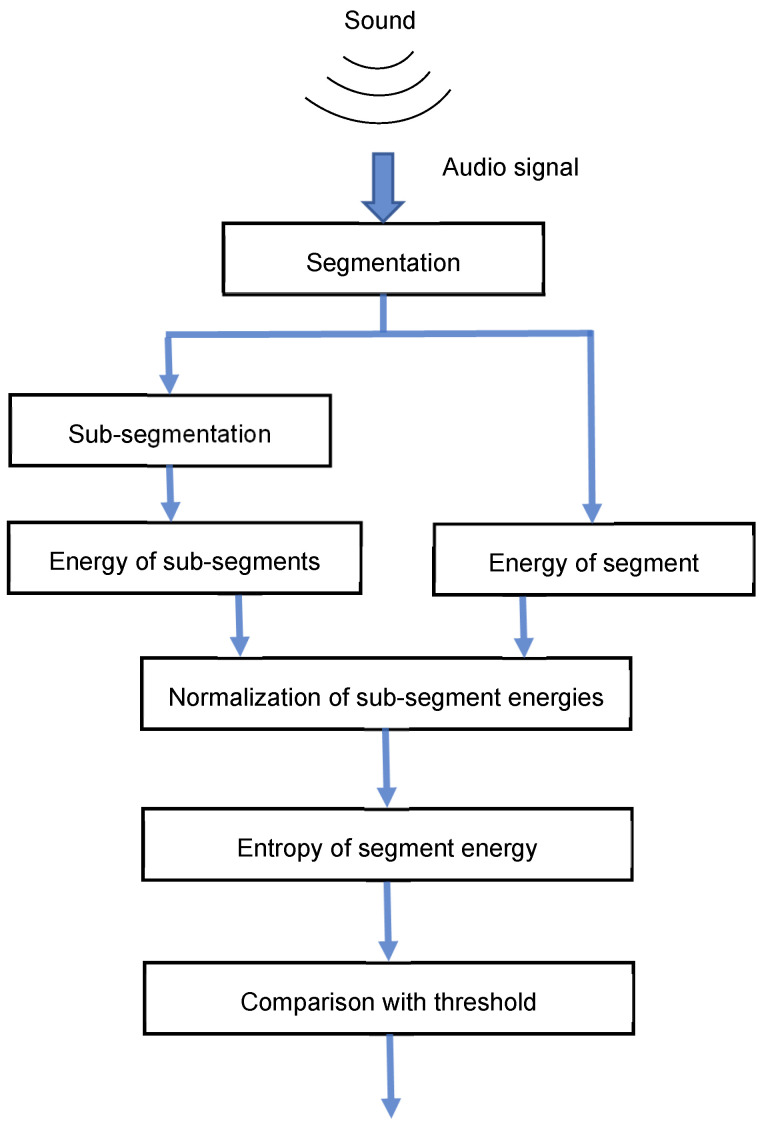
Block diagram of signal processing in the short-term entropy of signal energy.

The signal flow in the algorithm for gunshot detection based on the short-term energy entropy is depicted in the block diagram in Figure 6. Methods based on the principle of energy entropy are adapted by researchers in various applications for detection and recognition of specific signals. When examining electroencephalographic signals, energy entropies mapping four basic frequencies were extracted in [40] to improve the accuracy of brain wave classification, which can be employed, for example, in the early detection of drowsiness in drivers [41]. In underwater signal processing, the combination of energy entropy and wavelet decomposition achieved the highest recognition rate for four types of ship radiation signals [42]. In speech signal processing, a method for highly accurate detecting of the speech endpoints using logarithmic energy entropy of adaptive sub-bands was designed in [43].

## 4. Experimental Results and Discussion

The experimental analysis involves investigating the short-term entropy of the signal energy in various situations. The first series of experiments aimed to find the optimal combination of entropy parameters such as the segment size, number of sub-segments, logarithmization (ln vs. log), and, in addition, a suitable window type. The multi-parameter search was evaluated with respect to a reliable entropy threshold for gunshot detection. Based on the search results, the segment length was fixed at *N* = 880 samples, which represents a duration of 20 ms, each segment was divided into 10 sub-segments, logarithm naturalis (ln) was chosen, and the threshold value of 1.2 was set as the decision criterion for binary gunshot detection. These settings were used in further experiments.

**Figure 7:**
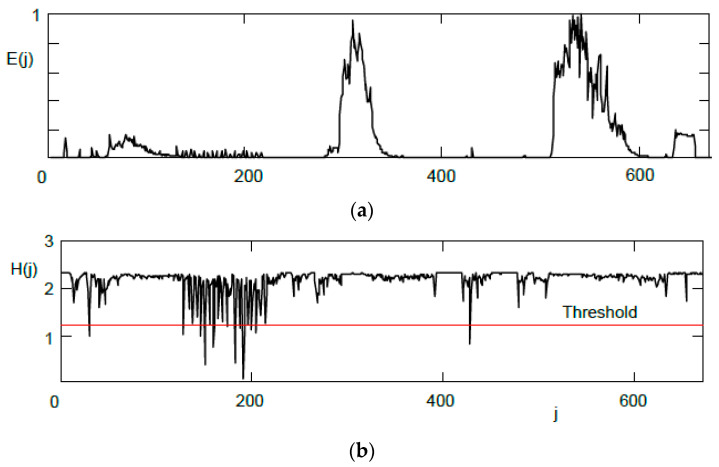
Short-term energy (**a**) and entropy (**b**) with marked threshold for gunshot detection.

Figure 7 illustrates the energy and entropy curves computed using a rectangular window without overlapping. The acoustic scene here includes two single gunshots, one burst, a barking dog, a snorting elephant, and a car horn at the end. The loudest sounds are from elephants (twice). In places where the entropy curve *H*(*j*) falls under the threshold line, gunshots are detected. As can be seen, the single gunshots are reliable detected, but not all individual gunshots in the burst are correctly identified. This phenomenon also occurs in other test signals with a burst. However, such inaccuracy is not considered problematic because the burst is detected as a whole.

For comparison, out of curiosity, we fed the signals of six synthetic gunshots intended for fun to the input of the algorithm. These signals were of normal intensity and all were correctly recognized as non-gunshots.

### 4.1. Evaluation Metrics

For assessing the performance of the proposed algorithm when weak gunshots and loud other sounds occur, we use a power comparison called the gunshot-to-sound ratio (GSR), which is defined in decibels [dB] as

(11) $$GSR=10\;\log_{10}\frac{G}{S_{\mathrm{aver}}},$$

where *G* is the power of a gunshot (i.e., the power of a short segment containing the gunshot) and *S*_aver_ is the average power estimated from non-gunshot sounds over the test audio signal. Thus, the calculation of GSR is independent of the type of non-gunshot sounds surrounding the gunshot, as well as the length of silence intervals.

In addition to impulsive non-gunshot sounds, the performance of the algorithm was also tested in a continuous noise environment, which can be characterized by the power relationship between noise and gunshots as the gunshot-to-noise ratio (GNR) expressed in decibels [dB] as

(12) $$GNR=10\;\log_{10}\frac{G}{N},$$

where *G* is the power of gunshots and *N* is the long-term noise power estimated using the standard deviation over the whole noise signal.

To evaluate the overall accuracy of the detection approach, two standard metrics were used—recall, also termed sensitivity or the true positive rate, and precision, also termed the positive predictive value. These metrics are defined as percentages as follows

(13) $$Recall=\frac{TP}{TP+FN}\cdot 100,$$

(14) $$Precision=\frac{TP}{TP+FP}\cdot 100,$$

where *TP* is the number of true positives (gunshots recognized as gunshots), *FN* is the number of false negatives (gunshots identified as non-gunshots, i.e., ignored gunshots) and *FP* is the number of false positives (non-gunshots mistaken for gunshots). These metrics were chosen considering that they do not contain true negatives, which represent much more acoustic events than the number of TPs in the real-world. The higher *Recall* and *Precision* values reflect a better performance of the algorithm. Ideally, they achieve 100%.

### 4.2. Overall Detection Accuracy with Ambient Impulsive Sounds

In order to optimize the sound signal processing, we preliminarily investigated the effect of overlapping adjacent signal segments on the reliability of gunshot detection on a relatively small amount of data. In these tests, it was observed whether each gunshot is actually detected as a gunshot. The test signals contained counterbalanced groups of 100 single gunshots and 100 non-gunshots consisting of a dog barking, an elephant snorting, and a car horn. The overlap was gradually set to 10, 20, 30, 40, 50, and 60 percent of a fixed segment length of 20 ms. Based on the experimental results, the best overlap size appears to be 50 percent. In this case, the time overlap is 10 ms and the shift of the analyzed segment is also 10 ms. Table 1 shows a comparison of the achieved detection rate for a 0% and 50% overlap at different GSR levels. A rectangular window was used in these tests. The improvement when applying a 50% overlap is clearly seen for moderately weak gunshots in the GSR range between −4 dB and −10 dB.

The proposed algorithm was further tested using more diverse sounds. Here, the group of gunshots consists of 510 single gunshots and 62 bursts of different lengths from 5 to 18 individual gunshots in one burst. The non-gunshot group includes short and long dog barks, various elephant sounds, natural thunder, splashing water, breaking branches, a car horn, and human screams—a total of 585 sounds. In the binary detection of weak gunshots, the GSR levels were gradually decreased until detection failed completely. At each GSR level, the same input data were processed once using a rectangular window and once using a Hamming window. The achieved results in terms of *Recall* and *Precision* are shown in Table 2 without segment overlapping and in Table 3 with 50% overlapping.

**Figure 8:**
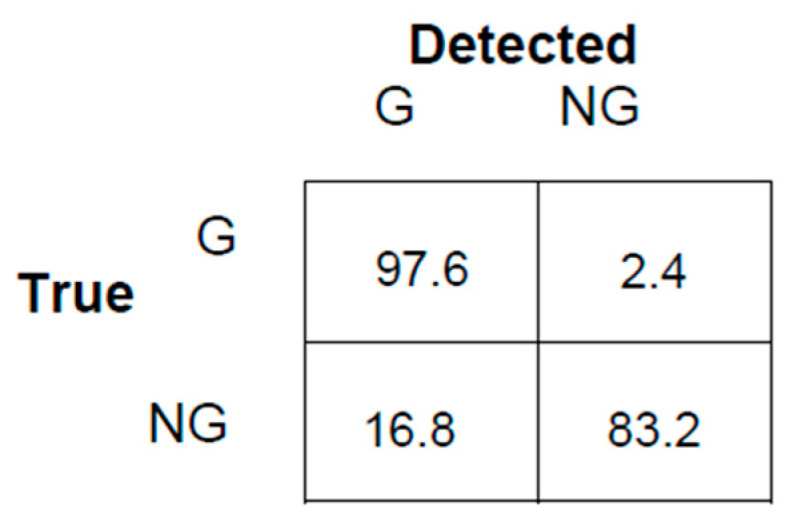
Confusion matrix for −6 dB with rectangular window; G: gunshots, NG: non-gunshots.

Comparing the values of *Recall* and *Precision* on each row in Table 2 and Table 3, the rectangular window comes out more efficient at each GSR level. In addition, the gunshot detection using a Hamming window can partially work only up to −8 dB. In summary, with a rectangular window of length 20 ms shifted by 10 ms, a good detection efficiency was achieved for GNR ≥ −8 dB and a few gunshots were detected up to −13 dB. An example of the distribution of correct/wrong detection is mapped by the confusion matrix in Figure 8.

### 4.3. Overall Detection Accuracy with Background Noises

**Figure 9:**
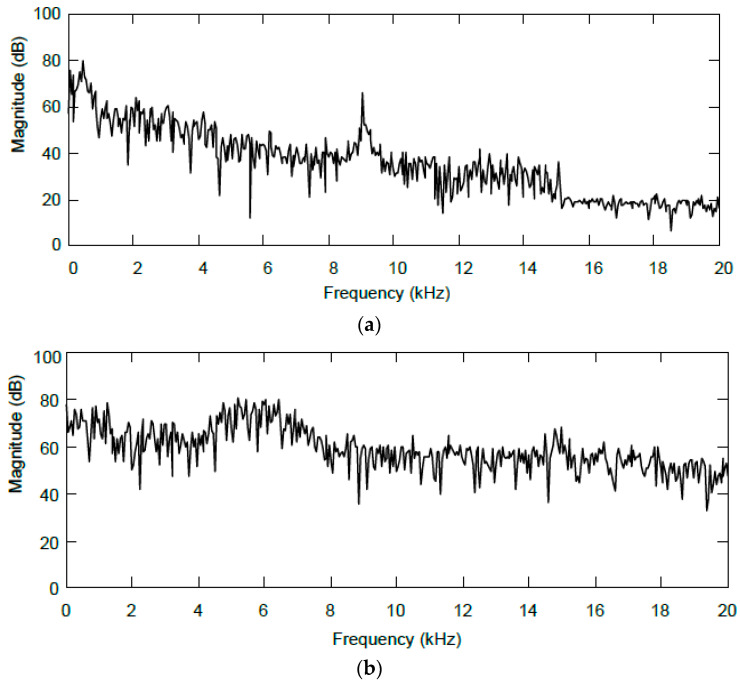
Spectra of real noise signals used: (**a**) flying helicopter; and (**b**) factory noise.

In another series of tests, three different types of continuous non-impulsive noise were used. The first one is synthetically generated Gaussian noise with a zero mean and a standard deviation of 1.0, having a balanced spectral envelope resembling white noise. The second noise is the recorded sound of a low-flying helicopter, which has an approximately linearly decreasing spectral envelope with one distinct narrow local maximum around the tone at 9044 Hz. This frequency is determined by the technical design of the helicopter. The third noise is a recorded factory noise with one broad local maximum in the spectrum. Figure 9 shows the spectra. All noises used can be considered stationary.

Each noise signal was mixed with short gunshot signals, with all test gunshots individually having the same power. The power of the noise signal can be controlled to achieve the desired GNR level. At different GNR levels, the detection results were evaluated by means of *Recall* and *Precision*. A total of 60 individual gunshots were tested in background noise. At one GNR level, each gunshot was tested three times at three randomly set positions in the noise; i.e., the result listed in each cell in the following tables was computed from 180 individual gunshot tests. All results presented here were obtained using a rectangular window. Other types of windows have proven to be less effective for handling continuous noise.

**Figure 10:**
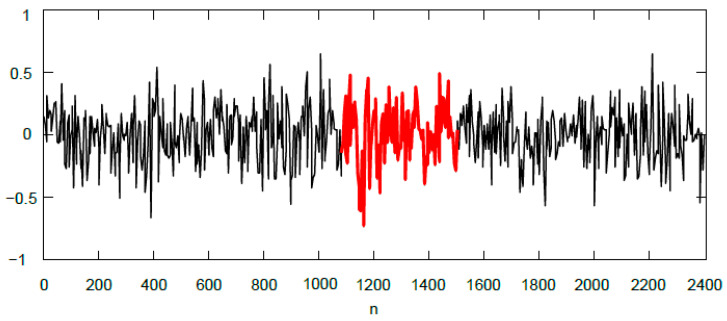
Background noise signal (black line) with gunshot (bold red line) detected at GNR = −12 dB.

In the case where binary gunshot detection is based on an entropy threshold level, the threshold value should be adapted to the intensity and nature of the noise (assuming the noise is stationary). The decision threshold in our tests was experimentally determined in the training process for each type of continuous noise based on the inverse entropy peaks. The results obtained with two different threshold settings derived from less loud (GNR = 0 dB) and louder (GNR = −10 dB) Gaussian noise are shown in Table 4 and Table 5, respectively. Similar results obtained for helicopter noise are shown in Table 6 and Table 7, as well as for factory noise in Table 8 and Table 9. Outside the GNR range presented in the tables, gunshot detection works flawlessly in low noise at GNR > 5 dB, while, in high noise at GNR < −13 dB, all gunshots remain undetected. The lowest GNR at which some gunshots can still be detected is −13 dB. Figure 10 shows the noise signal along with the weak gunshot in the time domain. In this case, the gunshot is drowned in Gaussian noise (GNR = −12 dB) and the algorithm is able to distinguish it from the background noise.

As can be seen, factory noise and helicopter noise make gunshot detection more difficult than Gaussian noise. The detection is most affected by the sound of the helicopter, particularly at low GNR levels. In summary, the results in Table 4, Table 5, Table 6, Table 7, Table 8 and Table 9 show that the decision threshold derived from GNR = −10 dB is more effective for detecting weak gunshots than the threshold derived from GNR = 0 dB. Using this threshold, a good detection rate was achieved for GNR ≥ −6 dB. When the GNR further deteriorates, the detection rate drops significantly.

### 4.4. Comparison with Other Methods

A direct comparison of the proposed method with other authors’ methods for the detection of weak gunshots is not very feasible due to the lack of reports specifically focused on weak gunshots. For reference, Table 10 shows some results of state-of-the-art methods and systems developed for the detection of common gunshots. The authors of the referred publications do not provide any information about the signal-to-noise ratio. It can be assumed that the tested gunshots and non-gunshots have intensities of approximately the same order. The studies listed in Table 10 belong to two application categories. The first is focused only on recognizing gunshots [7,8,44,45]. Here, the algorithms are optimized exclusively for gunshot signals, and the signal processing leads to binary decisions. The systems in the second category are focused on the joint recognition of typical acoustic events in the interest of safety, such as breaking glass, gunshots, screams, and children crying [30,46,47,48]. A somewhat unusual group of acoustic events for recognition is presented in [47], consisting of music, speech, gunshots, beatings, and screams. Study [48] reports measurements in 15 different background scenes. The most similar scene to our environment is the beach. Therefore, the results obtained on the beach were used.

All systems in the second category (lower half of Table 10) have gunshot detection as one of the goals in multi-class signal classification. In these cases, the recall and precision values presented are for the gunshot class, not averaged over all recognized classes. In general, the best result from each study listed in Table 10 was always taken into account for comparison (if multiple results are available).

Perhaps the closest comparison to weak gunshots can be made with study [35]. The authors measured and controlled the energy of the sounds of interest and background noise and investigated the effect of SNR on recognition based on spectrograms and MFCCs. The sounds of interest include three classes, namely, gunshots, glass breaking, and screams. Background noise contains Gaussian noise and a variety of indoor and outdoor audio signals such as applause, claps, bells, rain, whistles, etc. The system was trained and tested at different SNR levels, ranging from −5 dB to 30 dB with a step of 5 dB. The cases of low SNR levels, i.e., 0 dB and −5 dB for both training and testing, are compared with our results in Table 11. In the row Proposed method, the results from Table 4 and Table 5 obtained with Gaussian noise are used. Other comparable results can be found in Table 6 and Table 7 (helicopter noise) and Table 8 and Table 9 (factory noise).

Apart of the performance comparison by *Recall* and *Precision*, the proposed method has the advantage of a lower computation complexity and memory requirements than other methods. In fact, it is based on only one feature, i.e., energy entropy, while other methods used a set of conventional features. For example, the study with the best published results [30] is based on the widespread MFCCs, which consist of 40 extracted basic coefficients and a total of 240 features derived from them.

## 5. Conclusions

This paper deals with the detection of weak hunting gunshots using the short-term entropy of signal energy computed from acoustic signals that are captured in surrounding of shots in the wild. Our research in this area was primarily aimed at detecting gunshots of the usual intensity to protect wild elephants from poachers—the suppliers of illegal ivory markets. In our past research, we used various features for this purpose, but not entropy. The core of the work presented here was to investigate the energy entropy to be optimized as a specific feature for the detection of weak gunshots. From this point of view, the decision strategy in the detection task was solved in a simple way using binary classification based on a threshold of the short-term entropy.

The proposed algorithm was explored for the detection of gunshots in two different categories of the ambient acoustic scene—short impulsive events and continuous background noise. In both categories, the algorithm was tested in power ratios between weak gunshots to louder surrounding sounds ranging from 0 dB to −14 dB. The overall accuracy was evaluated in terms of *Recall* and *Precision*. The experimental results show a high accuracy at levels from 0 dB to −2 dB and a satisfactory accuracy at levels from −2 dB to −6 dB. Then, the accuracy decreases as the gunshots are further attenuated, but some very weak gunshots can still be detected at −13 dB. A good effect of entropy can also be expected in the recognition of gunshots in an urban environment. Due to the relatively low computational complexity of the proposed algorithm, it could be easily integrated into existing detection systems and improve their reliability for common gunshots or extend their functionality to detect even more distant gunshots.

In summary, the main contributions of the work are as follows:

- A new feature effective for detecting weak gunshots was found—the short-term entropy of signal energy;
- The proposed algorithm was optimized and successfully tested for detection in two different kinds of acoustic scene: random series of impulsive sounds and continuous background noise;
- The detection of weak gunshots can extend existing detection systems to capture more distant gunshots;
- Due to the low computation complexity, the proposed method has the potential for effective real-time gunshot detection, as well as also for implementation in computation- and energy-constrained applications;
- No previous study has focused specifically on weak gunshots detection. This article covers a research gap regarding weak gunshots.

In future work, a natural next step is to develop a procedure for automatically determining the adaptive decision threshold in different acoustic environments. Furthermore, the research will continue by analyzing the energy entropies of signals in various frequency sub-bands. A flexible filter bank should automatically adapt to the current background noise. Depending on the noise characteristic, the energy entropy from the optimal sub-band or a combination of energy entropies from selected sub-bands could yield new useful results. For these purposes, further real-world acoustic scenes must be included in the research. It will also be useful to explore the possibilities of the proposed method using AI.

## Figures and Tables

**Table 1:** Effect of segment overlapping on percent detection rate at different GSR levels.

GSR (dB)	Non-Overlapping	Overlapping 50%
0	100	100
−2	100	100
−4	92	100
−6	92	100
−8	58	83
−10	17	33
−12	14	14
−13	5	6
−14	0	0

**Table 2:** Gunshot detection score (in percent) for different windows without overlap.

GSR (dB)	Rectangular		Hamming	
	Recall	Precision	Recall	Precision
0	100	93.3	100	91.1
−2	99.7	92.2	99.3	87.8
−4	91.6	91.4	66.4	80.9
−6	91.1	91.2	41.4	71.8
−8	58.2	86.5	8.0	33.1
−10	20.8	66.1	0	0
−12	16.4	60.3	0	0
−13	8.2	42.7	0	0
−14	0	0	0	0

**Table 3:** Gunshot detection score (in percent) for different windows with 50% overlap.

GSR (dB)	Rectangular		Hamming	
	Recall	Precision	Recall	Precision
0	100	93.2	100	50.3
−2	100	92.9	97.9	49.4
−4	98.3	85.7	91.8	47.6
−6	97.6	85.1	65.6	39.2
−8	83.7	83.0	7.2	6.6
−10	34.1	57.2	0	0
−12	16.6	39.6	0	0
−13	8.4	24.6	0	0
−14	0	0	0	0

**Table 4:** Gunshot detection in Gaussian noise (threshold derived from GNR = 0 dB).

GNR (dB)	Recall (%)	Precision (%)
5	100	100
0	100	100
−2	99.4	99.4
−4	96.7	99.4
−6	91.1	98.8
−8	60.6	97.3
−10	11.7	87.5
−12	1.7	50.0
−13	0	0

**Table 5:** Gunshot detection in Gaussian noise (threshold derived from GNR = −10 dB).

GNR (dB)	Recall (%)	Precision (%)
5	100	100
0	100	100
−2	99.4	97.3
−4	99.4	96.8
−6	97.8	96.2
−8	75.0	95.1
−10	27.2	90.7
−12	8.3	71.4
−13	3.9	72.2

**Table 6:** Gunshot detection in helicopter noise (threshold derived from GNR = 0 dB).

GNR (dB)	Recall (%)	Precision (%)
5	99.4	80.3
0	61.1	72.8
−2	30.6	47.4
−4	12.8	27.7
−6	9.4	23.3
−8	8.9	22.2
−10	6.7	18.5
−12	4.4	12.3
−13	2.8	7.7

**Table 7:** Gunshot detection in helicopter noise (threshold derived from GNR = −10 dB).

GNR (dB)	Recall (%)	Precision (%)
5	100	100
0	100	100
−2	97.8	100
−4	95.6	98.9
−6	85.6	98.7
−8	48.9	97.8
−10	20.6	92.5
−12	6.1	73.3
−13	2.8	45.5

**Table 8:** Gunshot detection in factory noise (threshold derived from GNR = 0 dB).

GNR (dB)	Recall (%)	Precision (%)
5	100	100
0	100	100
−2	98.9	100
−4	97.2	99.4
−6	87.2	99.4
−8	46.7	97.7
−10	14.4	89.7
−12	3.9	70.0
−13	3.3	66.7

**Table 9:** Gunshot detection in factory noise (threshold derived from GNR = −10 dB).

GNR (dB)	Recall (%)	Precision (%)
5	100	100
0	100	100
−2	100	100
−4	97.2	99.4
−6	87.2	98.7
−8	50.0	97.8
−10	20.6	92.5
−12	6.7	75.0
−13	3.3	54.5

**Table 10:** Results of previous studies with SNR > 0 dB; LPC: linear predictive coding, MFCC: mel-frequency cepstral coefficient, GFCC: gammatone frequency cepstral coefficient.

Reference	Features	Recall (%)	Precision (%)
[7]	Mel spectrograms	98	87
[8]	Spectrogram images	95	85
[44]	MFCC, discrete wavelet transform	97	97
[45]	Mel spectrograms	95	93
[30]	MFCC, Δ, ΔΔ	100	98
[46]	LPC, MFCC, GFCC	94	76
[47]	MFCC, spectrograms, spectral roll-off, chroma, entropy, zero-crossing rate	79	71
[48]	MFCC, spectral centroid, spectral roll-off, zero-crossing rate, energy	95	96

**Table 11:** Comparison of results with previous study with SNR ≤ 0 dB.

Method	Training/Testing	Recall (%)	Precision (%)
Ioannis Papadimitriou et al. [35]	0 dB/0 dB	88	89
	−5 dB/−5 dB	79	82
Proposed method	0 dB/0 dB	100	100
	−10 dB/−6 dB	98	96

## Data Availability

The original contributions presented in the study are included in the article, further inquiries can be directed to the corresponding author.
